# Spino‐Pelvic Alignment and Surgical Outcomes in Chinese Elderly With Lumbar Stenosis Syndrome: A Prospective Study

**DOI:** 10.1002/agm2.70036

**Published:** 2025-08-16

**Authors:** Chong Zhao, Linyu Jin, Yan Liang, Haiying Liu, Shuai Xu

**Affiliations:** ^1^ Department of Spinal Surgery Peking University People's Hospital, Peking University Beijing People's Republic of China; ^2^ Department of Orthopedics, Shanghai Key Laboratory for Prevention and Treatment of Bone and Joint Diseases, Shanghai Institute of Traumatology and Orthopedics Ruijin Hospital, Shanghai Jiaotong University School of Medicine Shanghai China

**Keywords:** biomechanics of spino‐pelvic interaction, lumbar stenosis syndrome (LSS), spino‐pelvic sagittal alignment, SRS‐Schwab classification

## Abstract

**Objective:**

This study aimed to systematically identify and quantify the acceptable spino‐pelvic parameters for Chinese elderly patients with lumbar stenosis syndrome (LSS) following surgical treatment.

**Method:**

A prospective study of 165 LSS cases from July 2018 to June 2020 evaluated spino‐pelvic alignment using PI, LL, PT, and SVA, categorized by SRS‐Schwab classification. Quality of life was assessed via the Oswestry disability index (ODI), and parameter thresholds were determined by AICc and ROC analysis.

**Results:**

No significant change in radiological parameters was observed postsurgery. However, clinical outcomes improved with lower grades of PI‐LL and PT. The mean threshold for PI‐LL and PT at baseline was 18.8° and 26.1°, respectively. At the endpoint, PI‐LL was 17.7°, and PT was 26.1°, indicating optimal ranges for surgery.

**Conclusion:**

Spino‐pelvic alignment significantly correlates with quality of life in elderly LSS patients. The study suggests that PI‐LL and PT should be maintained within 17.7° and 26.1°, respectively, for optimal surgical outcomes.

## Graphical abstract

This study highlights the critical role of maintaining spino‐pelvic alignment in elderly patients with lumbar spinal stenosis. Postsurgical thresholds for PI‐LL (17.7°) and PT (26.1°) were associated with improved clinical outcomes, offering valuable benchmarks for surgical planning and postoperative care to enhance quality of life.

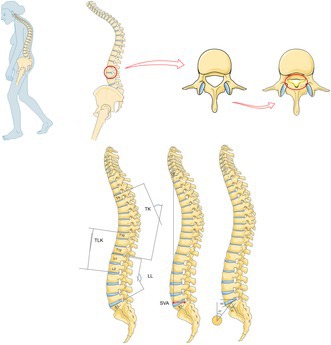

## 1 Introduction

Spino‐pelvic sagittal alignment plays a critical role in maintaining neurological function and quality of life. Efficient trunk adjustment is essential to balance the body's center of gravity over the feet, optimizing energy expenditure and reducing joint and muscle strain [1, 2]. Although the biomechanics of spino‐pelvic interaction and its parameter thresholds are well characterized, a universally accepted range remains elusive despite numerous theoretical frameworks [3, 4, 5].

The pelvis is fundamental to spinal sagittal alignment, with pelvic tilt (PT) serving as a key compensatory mechanism for maintaining balance [6, 7, 8]. Using the geometric relationship pelvic incidence (PI) = PT + sacral slope (SS), Schwab et al. established thresholds for three parameters linked to clinical outcomes: the difference between PI and lumbar lordosis (LL), PT, and the sagittal vertical axis (SVA) [9]. These parameters underpin the SRS‐Schwab classification for adult spinal deformity, which has achieved global consensus. However, the acceptable ranges of these parameters vary with age due to changes in lumbar alignment, pelvis realignment, and trunk muscle degeneration, underscoring the classification's non‐static nature [10].

Lumbar stenosis syndrome (LSS), a common condition among the elderly, often necessitates surgical intervention. Postsurgical quality of life in these patients depends not only on successful decompression and stable instrumentation but also on achieving appropriate spino‐pelvic alignment [11]. While the variability of these parameters across ages and populations is acknowledged, their quantification in elderly LSS patients, particularly postsurgery, remains inconsistent.

In Asia, and especially in China, less attention has been given to determining acceptable spino‐pelvic parameter ranges compared to Western studies [12, 13]. Given anatomical differences between Chinese and Western elderly populations, further research could yield valuable insights. This study, therefore, aims to investigate the relationship between clinical outcomes and spino‐pelvic alignment in Chinese elderly patients with severe LSS, utilizing the SRS‐Schwab classification. Additionally, it seeks to quantify the acceptable ranges of these parameters before and after surgery, addressing a critical gap in existing research.

## 2 Materials and Methods

### 2.1 Study Design and Ethics

This was a single‐center, prospective cohort study conducted at our institution from July 2018 to June 2020. Ethical approval was obtained from the institutional ethics committee (No. 2018PHC076), and all participants provided written informed consent. The study adhered to the STROCSS guidelines [14].

### 2.2 Patient Enrollment

#### 2.2.1 Inclusion Criteria

Participants were enrolled based on the following conditions:

1. Age and diagnosis: elderly patients aged 60 years or older with severe radiological and clinical lumbar spinal stenosis (LSS) (excluding L5/S1), unresponsive to at least 6 months of conservative treatment.
2. Spinal alignment: normal thoracic kyphosis (TK: 10°–40°), thoracolumbar kyphosis (TLK: −15°–15°), and lumbar lordosis (LL: 25°–53°); sagittal vertical axis (SVA) < 95 mm; and coronal alignment with a Cobb angle < 10°.
3. Radiological and clinical data: availability of pre‐ and postoperative whole‐spine radiographs, lumbar spine MRI, and clinical outcomes.

#### 2.2.2 Exclusion Criteria

Participants were excluded if they:

1. Had coronal or sagittal deformities, or had undergone prior extensive thoracolumbar surgery.
2. Were diagnosed with scoliosis, ankylosing spondylitis, Scheuermann's disease, or had a history of lumbar spine surgery.
3. Suffered from vertebral infections, spinal malignancies, severe comorbidities, or hip/knee disorders.
4. Belonged to younger age groups (< 60 years) or were lost to follow‐up.

### 2.3 Surgical Intervention

All patients underwent standard posterior lumbar interbody fusion (PLIF) surgery targeting the affected LSS levels. The upper instrumented vertebra (UIV) was maintained above L2, and the lower instrumented vertebra (LIV) was restricted to L5 to minimize pelvic rotation limitations. The surgeries were consistently performed by a senior surgeon to ensure procedural uniformity.

### 2.4 Spino‐Pelvic Parameters and Radiological Assessment

**FIGURE 1:**
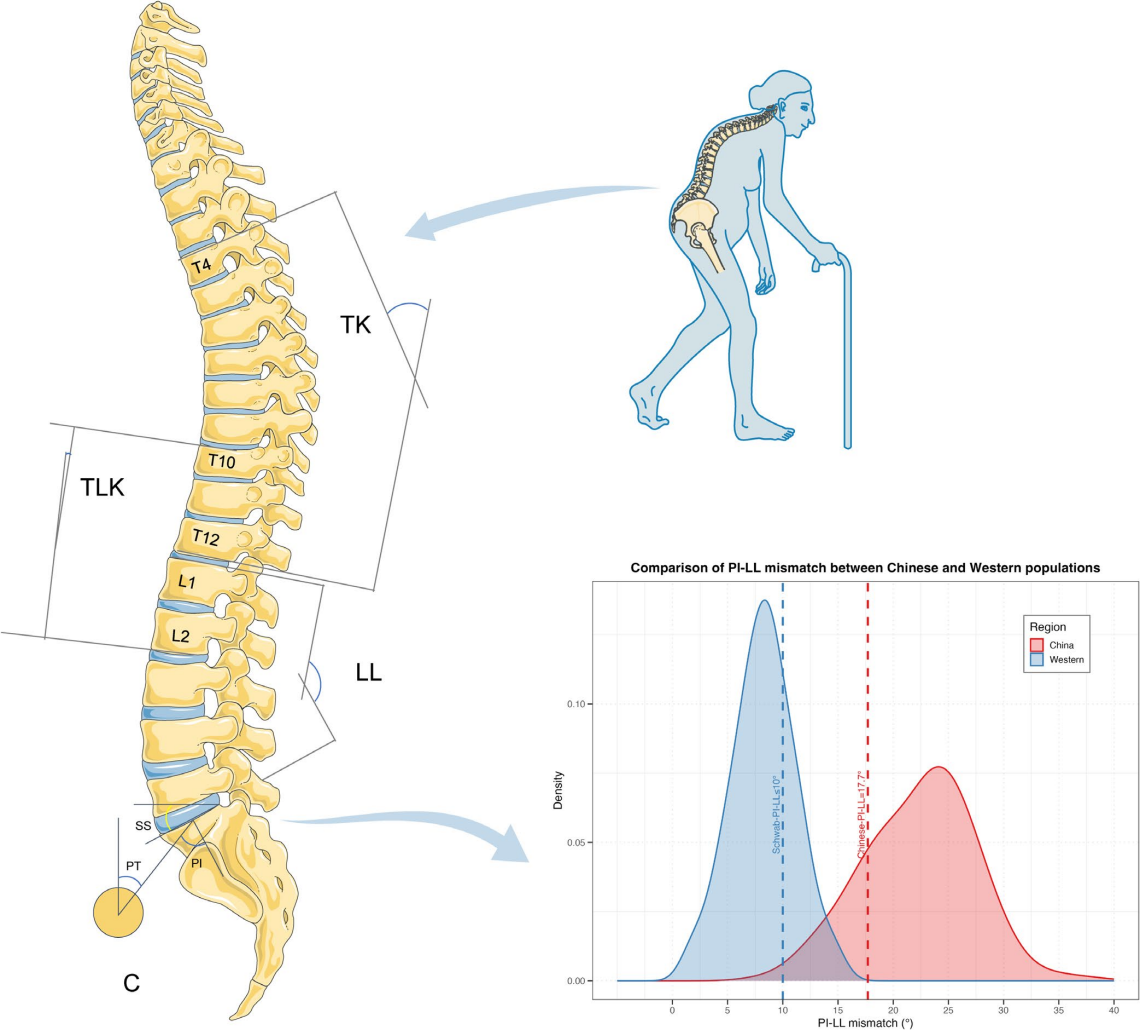
Schematic Representation of Spino‐Pelvic Sagittal Parameters. TK: Angle between T4 superior and T12 inferior endplates; TLK: T10 superior to L2 inferior endplates; LL: L1 superior to S1 superior endplates; PT: Vertical line‐femoral head center‐S1 superior midpoint; PI: S1 superior midpoint to femoral head line vs. vertical line. This study identified differences in these parameters between elderly Chinese LSS patients and Western populations.

As shown in Figure 1, radiological parameters were evaluated at baseline and the study endpoint using whole‐spine X‐rays. Measurements included thoracic kyphosis (TK), thoracolumbar kyphosis (TLK), lumbar lordosis (LL), sacral slope (SS), pelvic incidence (PI), and pelvic tilt (PT).

### 2.5 SRS‐Schwab Classification

Spino‐pelvic sagittal parameters—PI‐LL mismatch, PT, and SVA—were classified as follows*:

(*Footnote: Given the absence of coronal deformity, all cases were categorized as type “S” in the coronal curve typology.)

PI‐LL: Grade A: ≤ 10°; Grade B: 10°–20°; Grade C: > 20°.

PT: low (L): ≤ 20°; medium (M): 20°–30°; high (H): > 30°.

SVA: normal (N): ≤ 40 mm; protruded (P): 40–95 mm; very protruded (VP): > 95 mm.

### 2.6 Quality of Life Assessment

The Oswestry disability index (ODI) and visual analog scales (VAS) were employed to assess quality of life and pain intensity. ODI consisted of 10 questions with scores ranging from 0 to 50, where lower scores indicated better functional status. ODI was the primary clinical outcome measure. VAS was used to measure subjective pain levels on a scale of 0–10. Baseline ODI scores were categorized into four levels: mild, submild, subsevere, and severe dysfunction, while endpoint ODI scores were classified as excellent, good, fair, or poor based on quartiles.

### 2.7 Model Evaluation

Regression models were evaluated using the corrected Akaike's information criterion (AICc) to ensure accuracy in small sample scenarios. Models with the smallest AICc values were deemed optimal. Receiver operating characteristic (ROC) analysis was used, and the Youden index was calculated to determine the optimal cutoff for primary radiological parameters, which were validated using nonlinear regression.

### 2.8 Statistical Analysis

Paired Sample t‐tests were used to compare baseline and endpoint radiological and clinical parameters, and the analysis of variance (ANOVA) was applied to assess primary outcomes across SRS‐Schwab classification grades. Correlation analysis was performed using Pearson correlation and scatter plots to explore the relationships between radiological and clinical parameters, with further analysis conducted through linear regression models.

Data were expressed as mean ± standard deviation. Data analyses were conducted using SPSS 22.0 (IBM Corporation, Armonk, NY). A *p* < 0.05 was considered statistically significant.

## 3 Results

No statistically significant differences were observed in any radiological parameters at the endpoint compared to baseline; however, significant improvements were noted in VAS and ODI (*p* < 0.001) (Table 1). Initially, grade A of PI‐LL was the most common (58.8%), and the most frequent SRS‐Schwab classification was the lowest grade (35.8%). At the endpoint, the lowest grade decreased to 20.0%, while the middle grade (16.4%) and the moderate SVA group (13.9%) increased. Grade B of PI‐LL also increased to 41.2%. Additionally, one case showed grade VP of SVA with the SRS‐Schwab classification of moderate SVA (Table 2).

**TABLE 1:** The radiological and clinical parameters at baseline and endpoint of LSS group.

	Baseline	Endpoint	*p*
TK	26.9 ± 8.8	25.8 ± 10.3	0.252
TLK	6.8 ± 7.6	7.6 ± 7.6	0.077
LL	42.8 ± 12.5	41.6 ± 12.2	0.217
PI	51.7 ± 12.0	51.0 ± 11.0	0.208
PT	19.5 ± 8.8	19.4 ± 9.3	0.871
SS	31.9 ± 9.5	31.6 ± 9.6	0.701
SVA	26.6 ± 26.7	28.9 ± 31.4	0.705
PI‐LL	8.9 ± 12.0	7.9 ± 11.1	0.402
VAS	6.9 ± 1.2	1.7 ± 1.3	< 0.001*
ODI	37.7 ± 5.5	8.8 ± 7.8	< 0.001*

* *p* < 0.05.

**TABLE 2:** Different types at baseline and endpoint according to SRS‐Schwab classification.

	L		M		H	
	L‐Baseline	L‐Endpoint	M‐Baseline	M‐Endpoint	H‐Baseline	H‐Endpoint
A‐N	59	33	11	5	0	0
A‐P	19	23	8	11	0	1
A‐VP	0	0	0	0	0	0
B‐N	7	11	15	27	5	13
B‐P	3	4	5	4	2	8
B‐VP	0	1	0	0	0	0
C‐N	2	4	11	3	7	3
C‐P	0	3	7	2	4	9
C‐VP	0	0	0	0	0	0

*Note*: PI‐LL were graded as A (within 10°), B (10 ~ 20°) and C (> 20°); PT was graded as L (within 20°), M (20 ~ 30°), and H (> 30°); SVA was graded as N (within 40 mm), P (40 ~ 95 mm), and VP (> 95 mm).

At baseline, VAS was significantly correlated with PI‐LL (*p* = 0.027), while ODI showed correlations with both PI‐LL (*p* = 0.019) and PT (*p* = 0.001). At the endpoint, both VAS and ODI remained significantly correlated with PI‐LL and PT (*p* < 0.05). Significant differences in ODI and VAS were found across different PT grades in the SRS‐Schwab classification, with grade H scoring higher than grade L. At the endpoint, both ODI and VAS were most favorable in grade A of PI‐LL, followed by grades B and C (*p* < 0.001). Clinical outcomes were better in lower PT grades compared to higher grades, while no correlation was found between clinical outcomes and SVA (Table 3).

**TABLE 3:** Clinical outcomes by various grades of PI‐LL, PT, and SVA by SRS‐Schwab classification.

	I^a^	II	III	*p*
**ODI at baseline**				
PI‐LL	37.3 ± 5.6	37.6 ± 5.5	39.2 ± 4.9	0.240
PT	37.0 ± 5.6	37.7 ± 4.5	41.0 ± 6.0	0.009*
SVA	38.2 ± 5.7	37.3 ± 4.9	—	0.430
**VAS at baseline**				
PI‐LL	6.6 ± 1.3	6.8 ± 1.2	7.2 ± 1.4	0.061
PT	6.6 ± 1.2	6.9 ± 1.3	7.3 ± 1.5	0.035*
SVA	6.6 ± 1.3	6.7 ± 1.4	—	0.891
**ODI at endpoint**				
PI‐LL	5.1 ± 4.0	7.4 ± 3.8	17.1 ± 8.3	< 0.001*
PT	6.1 ± 4.2	7.6 ± 6.7	17.6 ± 9.6	< 0.001*
SVA	7.4 ± 4.8	9.2 ± 11.1	—	0.778
**VAS at endpoint**				
PI‐LL	1.2 ± 1.0	1.6 ± 1.0	3.0 ± 1.6	< 0.001*
PT	1.4 ± 1.0	1.7 ± 1.5	2.4 ± 1.1	0.013*
SVA	1.4 ± 1.1	1.5 ± 1.3	—	0.873

^a^ Various grades of PI‐LL, PT, and SVA, e.g., I meant Grade A of PI‐LL, Grade L of PT, and Grade N of SVA.

* *p* < 0.05.

**FIGURE 2:**
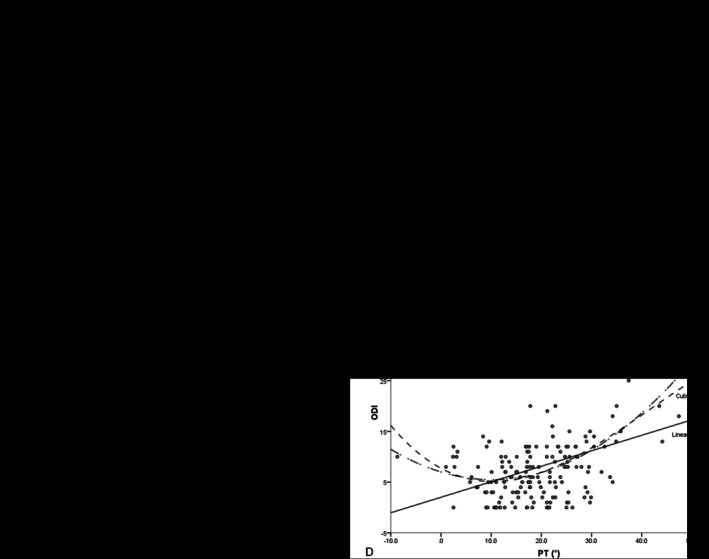
Non‐Linear Regression Analysis of ODI in Relation to PI‐LL and PT. (A) Baseline linear regression of ODI against PI‐LL. (B) Baseline linear regression of ODI against PT. (C) Endpoint cubic regression model for ODI and PI‐LL. (D) Endpoint quadratic regression model for ODI and PT.

The most appropriate regression models were identified for analysis. At baseline, a linear model showed that ODI correlated with PI‐LL (adjusted *R* ^2^ = 0.027, AICc = 560.4, *p* = 0.019), with the equation ODI = 0.083 × (PI‐LL) + 37.04. The same model also applied to the relationship between ODI and PT (adjusted *R* ^2^ = 0.056, AICc = 555.5), with ODI = 0.16 × PT + 34.78. At the endpoint, a cubic model was optimal for correlating ODI with PI‐LL (adjusted *R* ^2^ *= 0.687*, AICc = 449.1), while a quadratic model best described the relationship between ODI and PT (adjusted *R* ^2^ = 0.240, AICc = 534.9) (Table 4 and Figure 2). No significant correlation was found between ODI and SVA.

**TABLE 4:** Assessing PI‐LL, PT, or SVA for clinical outcomes using regression models (nonlinear regressions with best fit).

	Type of curve	F	*R* ^2^	Adjusted *R* ^2^	AICc	*p*
Baseline‐PI‐LL	Line^a^	5.596	0.033	0.027	560.4	0.019*
	Quadratic	2.786	0.033	0.021	562.5	0.065
	Cubic	1.849	0.033	0.015	564.6	0.140
Baseline‐PT	Line^a^	10.691	0.062	0.056	555.5	< 0.001*
	Quadratic	5.341	0.062	0.050	557.5	0.006*
	Cubic	3.551	0.062	0.045	559.6	0.016*
Baseline‐SVA	Line	0.045	0.000	−0.008	561	0.832
	Quadratic	1.563	0.026	0.009	559.6	0.214
	Cubic	1.186	0.030	0.005	562.0	0.318
Endpoint‐PI‐LL	Line	88.122	0.351	0.345	568.1	< 0.001*
	Quadratic	138.831	0.632	0.627	476.8	< 0.001*
	Cubic^a^	120.764	0.692	0.687	449.1	< 0.001*
Endpoint‐PT	Line	31.969	0.159	0.153	549.9	0.000*
	Quadratic^a^	25.600	0.250	0.240	534.9	< 0.001*
	Cubic	17.472	0.254	0.239	536.3	0.000*
Endpoint‐SVA	Line	1.231	0.032	0.006	684.1	0.274
	Quadratic	0.649	0.035	−0.019	694.3	0.528
	Cubic	0.434	0.036	−0.047	705.3	0.730

*Note: R* ^2^: the coefficient of determination.

^a^ Best fit of data with least error variance.

* *p* < 0.05.

**FIGURE 3:**
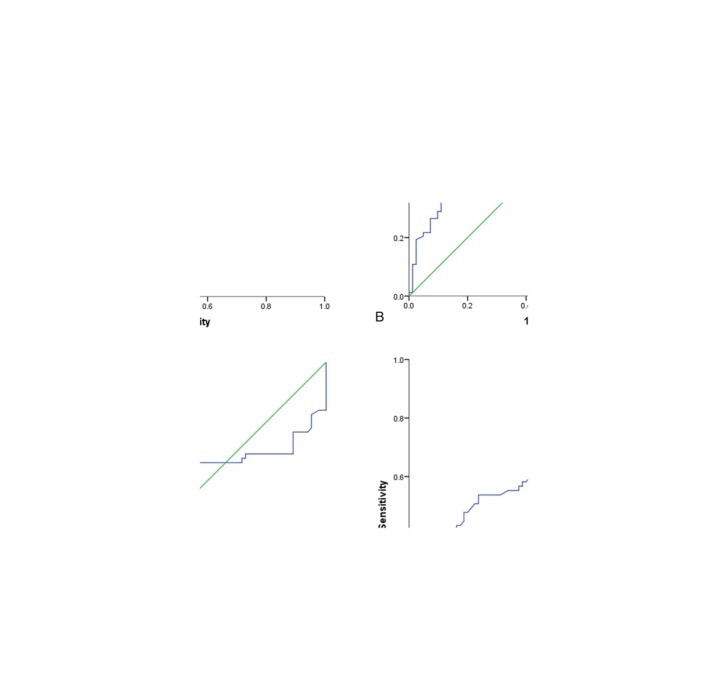
ROC analysis between ODI and PI‐LL or PT at baseline and the endpoint. (A) ROC was used to determine the cut‐off value of PI‐LL at baseline. (B) ROC was used to determine the cut‐off value of PT at baseline. (C) ROC was used to determine the cut‐off value of PI‐LL at the endpoint. (D) ROC was used to determine the cut‐off value of PT at the endpoint.

At baseline, the cutoff value of ODI distinguishing the lower and upper two quartiles was ODI = 39, with PI‐LL and PT calculated as 23.7° and 27.2°, respectively, based on the linear model. At the endpoint, ODI ranged from 0 to 40, and the cutoff value was ODI = 9. PI‐LL, determined by the cubic model, was 16.8°, while PT, determined by the quadratic model, was 26.2° (Table 5). ODI was treated as a dichotomous variable, with a cutoff of 39 at baseline and 9 at the endpoint. At baseline, the area under the curve (AUC) was 0.615, with PI‐LL corresponding to the ODI cutoff at 13.95° (*Youden Index = 0.226*) and PT at 25.05° (*AUC* = 0.667, Youden index = 0.264) (Figure 3), resulting in mean thresholds of 18.8° for PI‐LL and 26.1° for PT. At the endpoint, PI‐LL corresponding to ODI was 18.65° (*AUC* = 0.660, Youden index = 0.569), and PT was 26.00° (*AUC =* 0.622, Youden index = 0.299), yielding mean thresholds of 17.7° for PI‐LL and 26.1° for PT.

**TABLE 5:** The determination of the threshold of PI‐LL and PT by the cutoff value of ODI at baseline and the endpoint with the optimal nonlinear regression model.

	ODI interval	Corresponding ODI	PI‐LL (°)	SRS‐Schwab	PT (°)	SRS‐Schwab
Baseline	≤ 25%	[22, 33]	—	A	—	L
	25%–50%	(33, 39]	(0, 23.7]	A + B + C	(0, 27.2]	L + M
	50%–75%	(39, 41]	(23.7, 47.8]	C	(27.2, 40.1]	M + H
	75%–100%	(41, 50]	> 47.8	C	> 40.1	H
Endpoint	≤ 25%	[0, 3]	—	A	—	L
	25%–50%	(3, 9]	(−6.5, 16.8]	A + B	(−4.0, 26.2]	L + M
	50%–75%	(9, 15]	(16.8, 23.3]	B + C	(26.2, 35.9]	M + H
	75%–100%	(15, 40]	(23.3, +∞]	C	(35.9, 58.3]	H

## 4 Discussion

Spino‐pelvic alignment and balance significantly influence spinal function and serve as key guidelines for biomechanical reconstruction [15, 16, 17]. Schwab et al. identified the correlation between the lumbar spine and pelvis using the PI‐LL parameter, where a PI‐LL of ±10° was the threshold for a well‐aligned spino‐pelvic sequence. However, these thresholds can vary with age due to physiological degeneration, and this study highlights the importance of maintaining spino‐pelvic parameters within a certain range postsurgery for improved quality of life in elderly Chinese patients with lumbar spinal stenosis (LSS). While some studies suggest that fusion may not significantly improve outcomes in LSS patients, our findings emphasize that achieving optimal spino‐pelvic alignment—regardless of fusion status—correlates with better functional recovery. This highlights the potential role of alignment as an independent prognostic factor. To our knowledge, this is the first prospective study to establish spino‐pelvic parameter thresholds specifically for elderly Chinese LSS patients, addressing the paucity of Asian data in this field.

Lafage et al. established the relationship between age‐related spino‐pelvic parameters by enrolling 773 volunteers with an average age of 53.7 years [18]. Their study found that the ideal thresholds for these parameters increased linearly with age, from *PI‐LL* = 10.5°, *PT* = 10.9°, and *SVA* = 4.1 mm at age 35, to *PI‐LL* = 16.7°, *PT* = 28.5°, and *SVA* = 78.1 mm at age 75. They proposed an adjusted formula: *PI‐LL* = (age−55)/2 + 3, *PT* = (age−55)/3 + 20, and *SVA* = 2 × (age−55) + 25. To minimize age‐related heterogeneity, the study specifically targeted the elderly population, given the high prevalence of LSS in this group.

A Chinese study of 106 middle‐aged and elderly volunteers found that the spino‐pelvic pattern in the elderly differs from both the younger Chinese population and Western elderly, with a fitting relationship of LL = 0.6 PI + 0.4 TK + 10° [19, 20]. However, their data were not specific to elderly LSS patients and did not address the ideal postsurgery thresholds for parameters, highlighting the value of this study for further analysis.

In 2012, the Scoliosis Research Society (SRS) proposed the SRS‐Schwab classification for adult spinal deformity, which has become widely accepted [5, 21]. This system defines thresholds for PI‐LL, PT, and SVA across three grades. However, it was developed for Western populations, and the anatomical specificity of the Chinese spino‐pelvic structure warrants reconsideration. Additionally, the classification was designed for all age groups, not specifically for the elderly, and the static parameter boundaries may not align with the constantly adjusted optimal range. The SRS‐Schwab classification serves as radiological guidelines rather than clinical evidence, as patients with higher parameter grades may not necessarily experience worse quality of life. Zhang et al. found that, in spinal deformity patients undergoing long‐segment fixation, Grade B PI‐LL had lower ODI scores and a reduced rate of proximal junction kyphosis compared to Grades A and C [22]. In our study, both grades A and B were acceptable, with Grade A showing the lowest ODI, supporting Zhang's findings and suggesting that postsurgery parameter thresholds may be more flexible than Schwab's recommendations.

In our study, postoperative changes in the SRS‐Schwab classification (e.g., PI‐LL and PT changes) provide important insights for postoperative monitoring, surgical decision‐making, and personalized care. These changes reflect compensatory mechanisms in spino‐pelvic alignment, and monitoring them can help identify patients who may need further intervention. For instance, patients with an increased PI‐LL or PT postsurgery may require closer follow‐up to prevent negative impacts on functional recovery. Moreover, changes in SRS‐Schwab classification can optimize personalized care plans. Patients with higher PI‐LL or PT values may benefit from interventions like posture correction or physical therapy to improve alignment and reduce complications. Conversely, patients with better recovery may need less intensive monitoring, thus saving medical resources.

In terms of long‐term outcomes, changes in the SRS‐Schwab classification can help predict postoperative health trends [23]. Patients with lower postoperative classification grades may experience better functional recovery, while those with higher grades may face more spino‐pelvic‐related issues [24]. Adjusting follow‐up plans and interventions based on these changes can assist clinicians in predicting postoperative prognosis and adopting more tailored treatment strategies.

This study has some limitations that may affect the generalizability of its findings. Firstly, being a single‐center study, the results may not be fully representative of other populations, and multicenter research is needed for broader applicability. Besides, the short follow‐up period limits the ability to assess long‐term outcomes, and further studies with extended follow‐up are required. Lastly, this study is a single‐center trial with a relatively modest sample size (*n* = 165), which may limit the generalizability of our findings. Although we controlled for surgical variability by involving a single senior surgeon, future multi‐center studies with larger cohorts are needed to validate these thresholds across diverse populations.

## 5 Conclusion

In this study of elderly Chinese patients with LSS, significant improvements were observed in VAS and ODI scores (*p* < 0.001), although no major changes were seen in radiological parameters. The SRS‐Schwab classification shifted postsurgery, with an increase in middle and moderate SVA grades and Grade B of PI‐LL, while grade A of PI‐LL remained the most common. Both VAS and ODI scores correlated significantly with PI‐LL and PT at baseline and endpoint, with lower PT grades linked to better outcomes. The best clinical outcomes were associated with Grade A of PI‐LL, highlighting the importance of maintaining lower PI‐LL grades. No correlation was found between clinical outcomes and SVA.

Regression models indicated significant correlations between ODI and PI‐LL, with thresholds of 18.8° and 26.1° at baseline, and 17.7° and 26.1° at the endpoint, providing benchmarks for optimal spino‐pelvic alignment.

This study emphasizes the importance of maintaining specific spino‐pelvic parameters, especially PI‐LL around 17.7° and PT around 26.1° postsurgery, to enhance clinical outcomes in elderly LSS patients. These findings provide valuable insights for surgical planning and postoperative care, with potential applications in optimizing patient recovery. Future studies should explore the long‐term effects of spino‐pelvic alignment on functional recovery and quality of life.

## Author Contributions

**S.X**. designed the study. The manuscript was drafted by **C.Z**. and **L.J**., revised by **Y.L**. and **H.L**., **C.Z**., and **S.X**. were responsible for the data collection and the statistical analyses. All authors read and approved the final manuscript.

## Ethics Statement

All procedures performed in studies involving human participants were in accordance with the 1964 Helsinki declaration and its later amendments or comparable ethical standards, and this study was approved by the ethics committee of Peking People's Hospital (IRB approval: 2024PHB103‐001).

## Consent

All authors have confirmed that the content can be published and agreed to submit it for consideration for publication in the journal. Informed consent was obtained from all individual participants included in the study.

## Conflicts of Interest

The authors declare no conflicts of interest.

## Data Availability

The data used in this study are available from the corresponding author upon request.
